# Asiatic Acid Alleviates Myocardial Ischemia-Reperfusion Injury by Inhibiting the ROS-Mediated Mitochondria-Dependent Apoptosis Pathway

**DOI:** 10.1155/2022/3267450

**Published:** 2022-02-14

**Authors:** Chenlong Yi, Meijuan Song, Lifu Sun, Linjie Si, Dongmin Yu, Ben Li, Peng Lu, Wei Wang, Xiaowei Wang

**Affiliations:** ^1^Department of Cardiovascular Surgery, The Affiliated Hospital of Yangzhou University, Yangzhou University, Yangzhou, Jiangsu, China; ^2^Department of Cardiovascular Surgery, The First Affiliated Hospital of Nanjing Medical University, Nanjing, Jiangsu, China; ^3^Department of Respiratory and Critical Care Medicine, The First Affiliated Hospital of Nanjing Medical University, Nanjing, Jiangsu, China

## Abstract

Myocardial ischemia-reperfusion injury (MIRI) is a major cause of heart failure in patients with coronary heart disease (CHD). Mitochondrial dysfunction is the crucial factor of MIRI; oxidative stress caused by mitochondrial reactive oxygen species (ROS) aggravates myocardial cell damage through the mitochondria-dependent apoptosis pathway. Asiatic acid (AA) is a type of pentacyclic triterpene compound purified from the traditional Chinese medicine *Centella asiatica*, and its protective pharmacological activities have been reported in various disease models. This study is aimed at investigating the protective effects of AA and the underlying mechanisms in MIRI. To achieve this goal, an animal model of MIRI in vivo and a cell model of oxygen-glucose deprivation/reperfusion (OGD/R) in vitro were established. The results show that AA exerts a protective effect on MIRI by improving cardiac function and reducing cardiomyocyte damage. Due to its antioxidant properties, AA alleviates mitochondrial oxidative stress, as evidenced by the stable mitochondrial structure, maintained mitochondrial membrane potential (MMP), and reduced ROS generation, otherwise due to its antiapoptotic properties. AA inhibits the mitogen-activated protein kinase (MAPK)/mitochondria-dependent apoptosis pathway, as evidenced by the limited phosphorylation of p38-MAPK and JNK-MAPK, balanced proportion of Bcl-2/Bax, reduced cytochrome c release, inhibition of caspase cascade, and reduced apoptosis. In conclusion, our study confirms that AA exerts cardiac-protective effects by regulating ROS-induced oxidative stress via the MAPK/mitochondria-dependent apoptosis pathway; the results provide new evidence that AA may represent a potential treatment for CHD patients.

## 1. Introduction

Coronary heart disease (CHD) often leads to severe cardiac insufficiency, which is a significant challenge to human health. Sudden occlusion of coronary arteries during the ischemia phase causes tissue hypoxia and cellular adenosine triphosphate (ATP) depletion, and early reperfusion therapy to restore coronary blood flow after acute myocardial ischemia can save dying cardiomyocytes and prolong survival [1, 2]. However, rapid reintroduction of oxygen-rich blood to hypoxic myocardial tissues depleted of oxygen scavengers causes additional damage and aggravates cardiac dysfunction, known as myocardial ischemia/reperfusion (I-R) injury (MIRI) [3, 4]. MIRI is an important pathological factor leading to heart failure in patients with CHD that results in high morbidity and mortality rates [5, 6].

The premise of the molecular mechanism responsible for MIRI is complex and involves multiple cellular components [4, 7]. Oxidative stress, calcium overload, inflammatory response, and energy metabolism disorders have been shown to promote MIRI in various animal models. Among them, oxidative stress caused by an unregulated reactive oxygen species- (ROS-) mediated cascade during reperfusion is a key initiator of MIRI [1]. ROS broadly refers to a class of oxygen-containing compounds that are mainly derived from subsidiary products of the mitochondrial respiratory chain [8]. Under physiological conditions, ROS functions as a second messenger for signalling pathways that participate in the regulation of redox reactions in organisms and a variety of biological activities [1, 9]. On the contrary, excess ROS production will cause oxidative stress and damage [10]. A complex scavenging system network is established in the mitochondria to balance the endogenous production of ROS and subsequent oxidative stress [1]. However, pathological stimuli, including I-R injury, lead to a “burst” of ROS release from mitochondria that disrupts the endogenous antioxidant balance and ultimately leads to mitochondrial depolarization and mitochondrial outer membrane permeabilization (MOMP) and eventually cell death [11]. Hence, mitochondrial ROS have been a therapeutic target of interest for preventing I-R injury.

Asiatic acid (AA, C_30_H_48_O_5_) (Supplementary Figure A) is a type of pentacyclic triterpene compound purified from the traditional Chinese medicine *Centella asiatica* [12]. Its antioxidant, antiapoptotic, and antifibrotic pharmacological activities have been reported to exert an important protective effect on various disease models in the past decade [13–15]. In a rat model of middle cerebral artery occlusion (MCAO), AA restored the activity of antioxidant enzymes such as glutathione peroxidase and superoxide dismutase (SOD) in a dose-dependent manner, thus reducing the oxidative stress-induced damage to nerve function [16]. In a hepatic I-R injury model, the protective effect of AA was achieved by increasing the expression of peroxisome proliferator-activated receptor gamma (PPAR*γ*) and ultimately attenuating the activation of the NLR family pyrin domain containing 3 (NLRP3) inflammasome [17]. Pharmacological studies of AA in MIRI have also been documented [18, 19], but its mechanism remains ambiguous. In the current study, we combined in vivo and in vitro models to explore additional mechanisms of AA during MIRI and aimed to prove the potential pharmacological properties of AA in the clinical treatment of CHD.

## 2. Materials and Methods

### 2.1. Materials

Purified natural extracts of AA (97%) and dimethyl sulfoxide (DMSO) were purchased from Merck KGaA. SB203580 and SP600125 were purchased from Merck KGaA. Dulbecco's modified Eagle medium (DMEM) and fetal bovine serum (FBS) were purchased from Gibco (Thermo Fisher Scientific, Inc.). The enhanced chemiluminescence detection substrate and restore western blot stripping buffer were purchased from Thermo Fisher Scientific, Inc. The cell counting kit-8 (CCK-8) assay kit was obtained from Dojindo Molecular Technologies, Inc. The total SOD assay kit, lipid peroxidation malondialdehyde (MDA) assay kit, BCA protein assay kit, and mitochondrial membrane potential (∆Ψm MMP) assay kit were purchased from Beyotime Institute of Biotechnology. All antibodies were purchased from Cell Signalling Technology, Inc. Unless indicated otherwise, all other chemicals and materials were purchased from Merck KGaA.

AA solution was freshly prepared as a stock solution in DMSO and diluted using saline to a final concentration. The vehicle control was prepared by mixing DMSO with normal saline (0.1% *v*/*v*).

### 2.2. Animals and MIRI Modelling

Male C57BL/6 mice aged 4-6 weeks and weighing 20-25 g were provided free access to food and water. All mice were housed at room temperature for 1 week for adaptation. For MIRI modelling, C57BL/6 mice were anesthetized and connected to an animal ventilator, and a limb-led electrocardiogram was recorded during modelling. Thoracic surgery was performed as previously described [20]. Briefly, a thoracotomy was performed on the left side of the chest between the third and fourth ribs to expose the left anterior descending (LAD) artery. During the ischemic phase, 7-0 Prolene was used to ligate the LAD artery. A plastic tube was passed through the incision to ensure the implementation of reperfusion after ligation surgery. The pale colour of the left ventricular anterior wall and obvious ST-segment elevation indicated the success of the operation. For the reperfusion phase, the tube was released, and the blood flow of the coronary artery was restored in 30 mins, followed by reperfusion for another 24 h.

**Figure 1:**
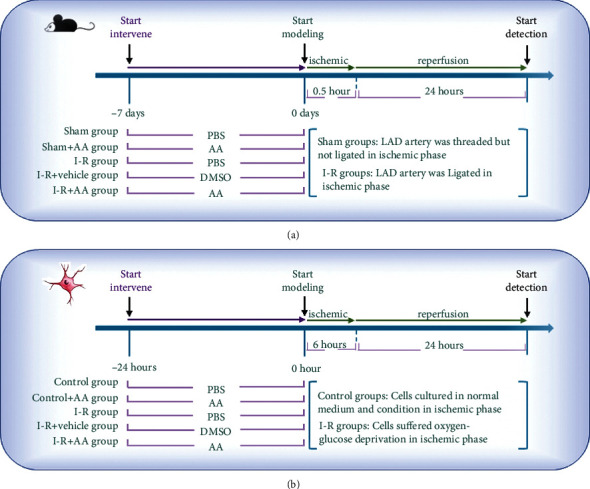
Summary of current experimental protocols. (a) Experimental grouping and protocols of in vivo experiment. (b) Experimental grouping and protocols of in vitro experiment. AA: asiatic acid; DMSO: dimethyl sulfoxide; LAD: left anterior descending artery; MIRI: myocardial ischemia/reperfusion injury; OGD/R: oxygen-glucose deprivation/reperfusion.

Five experimental groups were established for the in vivo experiments (Figure 1(a)). Before operation, C57BL/6 mice were randomly divided and given different gavage treatments for 7 days: (i) sham group: mice were pretreated with PBS (i.g.), (ii) sham+AA group: mice were pretreated with AA (100 mg/kg/day, i.g.), (iii) I-R group: mice were pretreated with PBS (i.g.), (iv) I-R+vehicle group: mice were pretreated with DMSO-solution (0.1% *v*/*v* DMSO, i.g.), and (v) I-R+AA group: mice were pretreated with AA (100 mg/kg/day, i.g.). During MIRI modelling, the LAD artery of mice in the sham groups (i+ii) was threaded but not ligated during the ischemic stage, while in mice in the I-R groups (iii-v), the LAD artery was ligated during the ischemic stage.

Animal care and the experimental protocols performed in the current study were approved by the Animal Care and Use Committee of Nanjing Medical University and implemented in accordance with the *Guidelines for the Care and Use of Laboratory Animals* published by the National Institutes of Health [21]. All efforts were made to minimize animal suffering.

### 2.3. Infarct Size Assessment

The myocardial infarct size post-MIRI was detected by performing Evans blue-2,3,5-triphenyltetrazolium chloride (EB-TTC) staining and measured as previously described [22]. Briefly, at the end of reperfusion, the LAD artery was reoccluded at the previous ligation site. One millilitre of 1% Evans blue solution was perfused retrogradely into the aorta to delineate the ischemic area from the nonischemic area. The hearts were excised, washed, frozen, and sliced into cross-sections. Afterward, the sections were incubated with a 1% TTC solution in the dark for 15 min at 37°C. The red part of tissues is the area at risk (AAR), and the white part is the area of infarct size (IS). After an overnight incubation with 4% paraformaldehyde, the sections were digitally photographed and areas were calculated (version 1.38x, National Institutes of Health, Bethesda, MD, USA). The myocardial infarct size was calculated as percentages of IS/AAR × 100%.

### 2.4. Echocardiography

Cardiac function was determined by performing echocardiography (Vevo 2100, Visual Sonics, Toronto, ON, Canada). Information on cardiac function was recorded before and 24 hours after modelling. Left ventricular function parameters were recorded, and the percentages of ejection fraction (%EF) and fractional shortening (%FS) were calculated.

### 2.5. Histological Analysis and Transmission Electron Microscopy (TEM)

At the end of modelling, heart samples were obtained.

For haematoxylin and eosin (H&E) staining, heart samples were fixed with 4% paraformaldehyde overnight and embedded in paraffin. Paraffin blocks were processed into 5 *μ*m thick sections. H&E staining was performed using standard protocols.

For TEM, heart samples were fixed with glutaraldehyde and citric acid. After dehydration with ethanol and acetone, tissues were embedded in epoxy resin. Ultrathin sections were prepared with a slicing machine and double-stained with uranium acetate and lead citrate. Ultrastructural changes in mitochondria in cardiac myocytes were observed under TEM as previously described [23].

### 2.6. Primary Cultures of Neonatal Rat Ventricular Myocytes (NRVMs)

Under aseptic conditions, newborn Sprague-Dawley rats aged 1-2 days were anesthetized with diethyl ether, and the heart was immediately removed and washed with PBS. After discarding the atria and aorta, the ventricles were minced and then digested with 0.1% type I collagenase and 0.125% trypsin. The released cells were collected by centrifugation and cultured in a 100 mm Petri dish in a humidified incubator with 5% CO_2_ at 37°C. After an incubation for 90 minutes, suspended NRVMs were harvested, seeded in 6-well culture plates at a density of 1 × 10^6^ cells/well, and cultured with high-glucose DMEM containing 10% FBS, 1% penicillin/streptomycin (*v*/*v*), and bromodeoxyuridine (BrdU, 100 *μ*M) for 48 h.

### 2.7. Oxygen-Glucose Deprivation/Reperfusion (OGD/R) Modelling

NRVMs in good condition were selected for modelling. The OGD/R model was established using a previously described method [24]. Briefly, an anoxic chamber (Mitsubishi Gas Chemical Company, Inc.) containing an Anaero-Pack was purchased to create a hypoxic atmosphere. The starved cells were cultured in low-glucose DMEM and placed in the hypoxic chamber to simulate the hypoxic-ischemic environment; at the same time, cells in the control groups were incubated with normal medium and conditions. After 6 hours, the medium of OGD-treated cells was replaced with complete medium, and cells were placed in a humidified 95% air-5% CO_2_ atmosphere at 37°C with cells in the control group for another 24 h of culture.

Five experimental groups were established for the in vitro experiments (Figure 1(b)). Before OGD/R modelling, NRVMs were homogenized and given different treatments for 24 h: (i) control group: cells were pretreated with PBS, (ii) control+AA group: cells were pretreated with AA (20 *μ*M), (iii) I-R group: cells were pretreated with PBS, (iv) I-R+vehicle group: cells were pretreated with DMSO (0.1% (*v*/*v*)), and (v) I-R+AA group: cells were pretreated with AA (20 *μ*M). During OGD/R modelling, cells in the control groups (i+ii) were cultured in normal medium and condition in the ischemic stage, while cells in the I-R groups (iii-v) were suffered oxygen-glucose deprivation in the ischemic stage.

### 2.8. Assessment of Cell Viability

The extracted NRVMs were cultured in 96-well plates. After exposure to the different treatments mentioned above, 10 *μ*L of CCK-8 solution was added to each well and incubated for 2 h in the dark. The absorbance was measured at 450 nm using a microplate reader (Bio-Rad Laboratories, Inc.). Cell viability is reported as the percentage of absorbance of each group relative to the control group.

### 2.9. Assessment of Oxidative Stress

Oxidative stress was measured by detecting the excessive production of MDA and decreased SOD activity. MDA content and SOD activity in mouse serum and cell supernatants were measured using assay kits according to the manufacturer's protocols and detected using a microplate reader (Bio-Rad Laboratories, Inc.).

### 2.10. Assessment of Intracellular ROS Generation

Dihydroethidium (DHE, Beyotime Institute of Biotechnology) staining was performed to detect ROS levels in myocardial tissues. Briefly, tissue sections were infused with the DHE working solution (10 *μ*M) in a humidified chamber at 37°C for 30 min. Intracellular ROS levels were detected under a fluorescence microscope (Nikon, Tokyo, Japan).

2′,7′-Dichlorofluorescein diacetate (DCFH-DA, Beyotime Institute of Biotechnology) staining was performed to detect ROS levels in NRVMs. Briefly, the supernatant was removed, and cells were washed. Then, the DCFH-DA working solution (10 *μ*M) was added to the cells and incubated for 30 min in the dark at 37°C. Fluorescence intensity was observed under a fluorescence microscope (Nikon, Tokyo, Japan).

### 2.11. Assessment of the MMP

Changes of MMP are an important feature of mitochondrial depolarization and MOMP, which precedes apoptosis [25]. After exposure to different treatments, NRVMs were washed with precooled PBS and incubated with the 5,5′,6,6′-tetrachloro-1,1′,3,3′-tetraethyl-imidacarbocyanine iodide (JC-1) working solution for 20 min according to the manufacturer's instructions. The plates were shaken every 5 min to ensure sufficient staining. Then, the stained cells were washed and visualized under a fluorescence microscope (Nikon, Tokyo, Japan). A decrease in the MMP is reflected as increased green fluorescence.

### 2.12. Assessment of Cell Apoptosis

For the detection of apoptosis, the TdT-mediated dUTP nick-end labelling (TUNEL) assay was performed in vivo and in vitro.

For the detection of myocardial tissues, paraffin-fixed left ventricular tissue sections were treated with xylene and a gradient of ethanol solutions. Subsequently, tissue sections were stained with both the TUNEL reaction mixture and Converter-POD for 1 h at 37°C according to the manufacturer's instructions. Then, stained sections were washed and visualized under a fluorescence microscope (Nikon, Tokyo, Japan).

For the detection of NRVMs, cells were fixed with 4% paraformaldehyde in PBS for 1 h at room temperature and then permeabilized with 0.1% Triton X-100 in 0.1% sodium citrate for 2 minutes on ice. Similarly, the cells were incubated with the TUNEL reaction mixture for 1 h at 37°C. After washing with PBS, cells were stained with DAPI for 5 minutes and analyzed under a fluorescence microscope (Nikon, Tokyo, Japan).

The apoptosis rate is reported as the percentage of the number of TUNEL-positive nuclei among the total number of nuclei.

### 2.13. Western Blot Analysis

Protein was extracted from myocardial tissue and cells according to the manufacturer's instructions. The protein concentration was measured using a BCA assay kit (Beyotime Institute of Biotechnology). Protein samples (30 *μ*g) were resolved on SDS-PAGE gels using electrophoresis and transferred to a PVDF membrane. After blocking with 5% skim milk for 90 min at room temperature, the membranes were washed with tris-buffered saline with Tween 20 (TBST) 3 times and incubated with the following primary antibodies (1 : 1,000) at 4°C overnight: anti-B-cell lymphoma-2 (Bcl-2), anti-Bax, anti-cytochrome c (cyt-c), anti-phosphorylated- (p-) p38, anti-p-JNK, anti-total p38, anti-total JNK, and anti-GAPDH rabbit polyclonal antibodies. After another 3 washes with TBST, the membranes were incubated with horseradish peroxidase-conjugated goat anti-rabbit secondary antibody (1 : 2,000) at room temperature for another 90 min. Then, the membranes were visualized using a chemiluminescence instrument and analyzed using ImageJ software. GAPDH was used as the internal reference.

### 2.14. Statistical Analysis

Data are presented as the means ± standard deviations. Statistical analyses were performed using GraphPad Prism version 9 (GraphPad Software, Inc.) and PASW Statistics 18.0 (SPSS Inc.). Comparisons between two groups were performed using two-way ANOVA and Tukey's post hoc tests. *P* < 0.05 was considered to indicate a statistically significant difference.

## 3. Results

### 3.1. AA Protects against Myocardial Damage following MIRI

**Figure 2:**
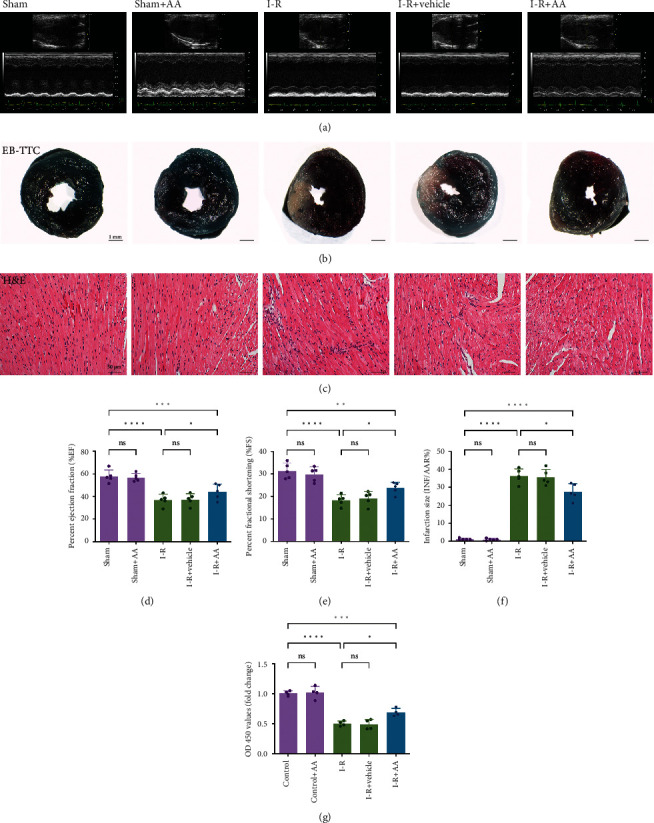
Effects of AA on cardiac function. (a) Representative transthoracic M-mode echocardiograms from each group following either MIRI or sham operation. (b) Infarction in left ventricle sections stained by EB-TTC (scale bars, 1 mm) (blue area: healthy tissue; red area: risk tissues; white area: infarct tissue). (c) Left ventricle sections stained by H&E (scale bars, 50 *μ*m). (d, e) Statistical analysis of %EF and %FS from mice. (f) Statistical analysis of the percentage of infarct volume was determined for each group. (g) The viability of cells was measured via the CCK-8 assay. Data represent the mean ± SD (^ns^*P* > 0.05, ^∗^*P* < 0.05,  ^∗∗^*P* < 0.01,  ^∗∗∗^*P* < 0.001, and^∗∗∗∗^*P* < 0.0001). EB: Evans blue; TTC: 2,3,5-triphenyltetrazolium chloride; H&E: haematoxylin and eosin; %EF: percent ejection fraction; %FS: percent fractional shortening; CCK-8: cell counting kit-8.

We investigated whether AA exerts cardioprotective effects on MIRI by measuring the cardiac function and infarct size of mice after different treatments. A significant reduction in cardiac function, including %EF and %FS, was observed in I-R mice (Figures 2(a), 2(d), and 2(e)). In addition, mice in the I-R group showed a larger proportion of myocardial infarct size (Figures 2(b) and 2(f)). However, no significant myocardial disorder or marked myocardial infarct was observed in the sham groups. Compared with the I-R group, pretreatment with AA significantly ameliorated these changes. Subsequently, we examined the improvement histologically using H&E staining, and the results showed that pretreatment with AA reduced the pathological changes in the myocardial tissue caused by MIRI (Figure 2(c)). In the in vitro study, the optimal AA concentration required for cell-based experiments was first confirmed. Therefore, we established treatment with a gradient of AA concentrations (0-100 *μ*M) and suggested that the activity of NRVMs was impaired when the concentration of AA exceeded 20 *μ*M (Supplementary Figure B). Therefore, 20 *μ*M AA was used in subsequent in vitro experiments. Based on the results from the CCK-8 assay, MIRI induced cell damage and suppressed cell viability, and the damage was attenuated by the AA intervention (Figure 2(g)). These data support the hypothesis that AA could improve cardiac function following MIRI.

### 3.2. AA Decreases Oxidative Stress following MIRI

**Figure 3:**
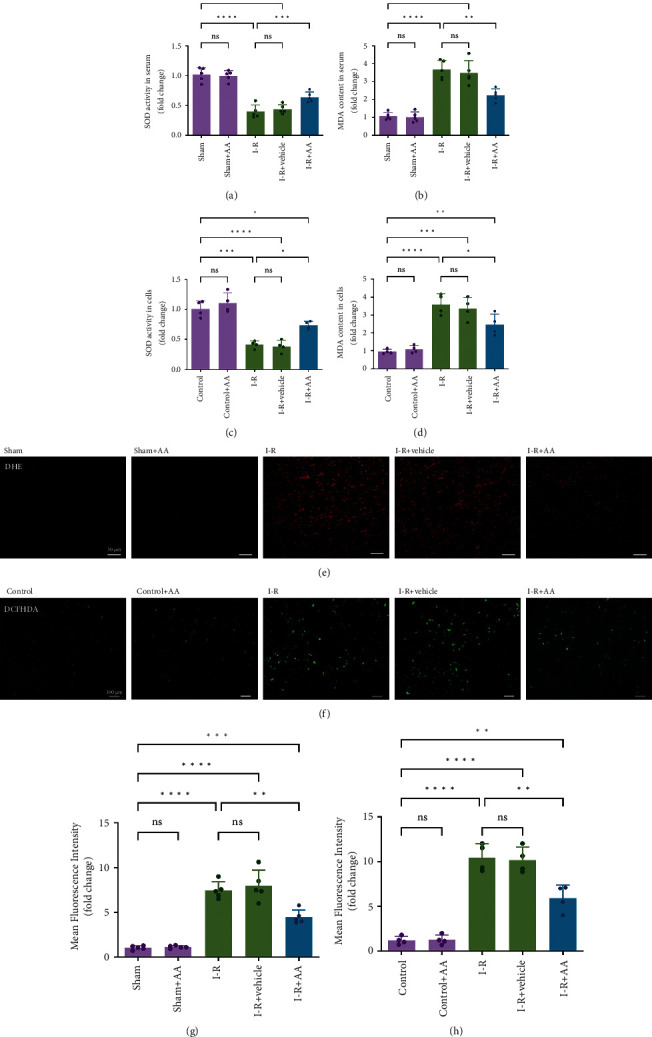
Effects of AA on oxidative stress. (a, b) Intracellular SOD activity and MDA production were measured in mouse serum. (c, d) Intracellular SOD activity and MDA production were measured in cell supernatants. (e) Intracellular ROS generation detection in heart tissues was photographed by DHE staining (scale bars, 50 *μ*m). (f) Intracellular ROS generation in cells was photographed by DCFH-DA staining (scale bars, 100 *μ*m). (g) Statistical analysis of ROS generation levels in tissues. (h) Statistical analysis of ROS generation levels in cells. Data represent the mean ± SD (^ns^*P* > 0.05, ^∗^*P* < 0.05,  ^∗∗^*P* < 0.01,  ^∗∗∗^*P* < 0.001, and^∗∗∗∗^*P* < 0.0001). ROS: reactive oxygen species; DHE: dihydroethidium; DCFH-DA: 2′,7′-dichlorofluorescein diacetate; SOD: superoxide dismutase; MDA: malondialdehyde.

We aimed to understand the effects of AA on oxidative stress induced by MIRI by measuring the levels of SOD and MDA in mouse serum and cell supernatants. MIRI increased the levels of MDA and reduced the levels of SOD in vivo and in vitro compared with the sham/control groups (Figures 3(a)–3(d)). AA pretreatment significantly increased SOD activity (Figures 3(a) and 3(c)) and, at the same time, decreased the increased MDA content (Figures 3(b) and 3(d)).

Unregulated ROS production is the main cause of oxidative stress, which promotes I-R injury [1]. Therefore, ROS production was determined in vivo and in vitro by performing DHE staining (Figure 3(e)) and DCFH-DA staining (Figure 3(f)), respectively. As shown, the DHE intensity in I-R-treated cardiac tissues was significantly increased compared with that in the sham-operated group (Figures 3(e) and 3(g)). Similarly, the fluorescence intensity of DCFH-DA in NRVMs exposed to OGD/R was also increased (Figures 3(f) and 3(h)), indicating ROS production during MIRI. However, AA pretreatment markedly reduced ROS production in cardiac tissue and cells. Taken together, these results suggest that the protective effects of AA on MIRI were associated with the alleviation of oxidative stress.

### 3.3. AA Preserves Mitochondrial Fission and Polarization during MIRI

**Figure 4:**
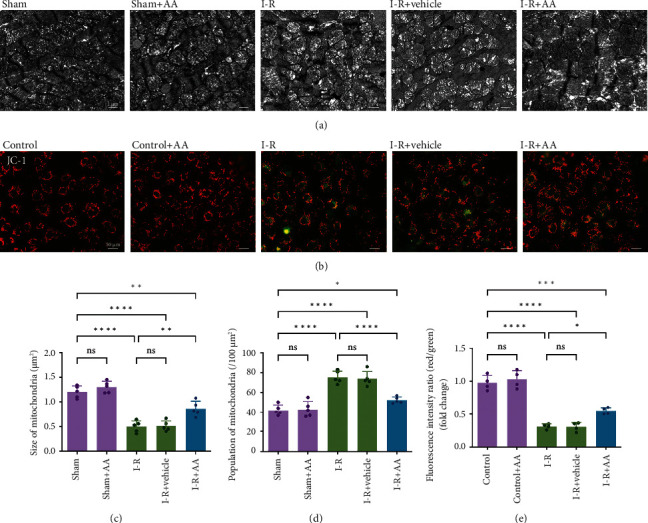
Effects of AA on mitochondrial damage. (a) Mitochondrial morphology in the myocardium was detected via transmission electron microscopy (scale bars, 1 *μ*m). (b) MMP in cells was determined by staining with the mitochondrial dye JC-1 (scale bars, 50 *μ*m). (c, d) Statistical analysis of physical sizes and the population sizes of the mitochondria from mice. (e) Statistical analysis of MMP changes in cells. Data represent the mean ± SD (^ns^*P* > 0.05, ^∗^*P* < 0.05,  ^∗∗^*P* < 0.01, and^∗∗∗∗^*P* < 0.0001). MMP: mitochondrial membrane potential (∆Ψm); JC-1: 5,5′,6,6′-tetrachloro-1,1′,3,3′-tetraethyl-imidacarbocyanine iodide.

Mitochondria are the main endogenous ROS source and target of damage [26]. According to previous studies, mitochondrial dysfunction is a vital component of I-R injury [5, 27, 28]. In our study, an electron microscope was used to observe the changes in mitochondrial morphology after I-R injury in vivo (Figure 4(a)). Correspondingly, JC-1 staining was performed to detect changes in the MMP after MIRI in vitro (Figure 4(b)). As shown in Figure 4(a), the mitochondria in the sham group had a regular shape and were arranged neatly, while the mitochondria showed fission and even vacuoles after MIRI (Figures 4(a), 4(c), and 4(d)). Meanwhile, MIRI caused the collapse of the MMP (Figures 4(b) and 4(e)), a sign of mitochondrial depolarization and MOMP, which herald the induction of cell apoptosis [25]. However, pretreatment with AA preserved the morphology and MMP of mitochondria to a certain extent.

### 3.4. AA Decreases Cell Apoptosis following MIRI

**Figure 5:**
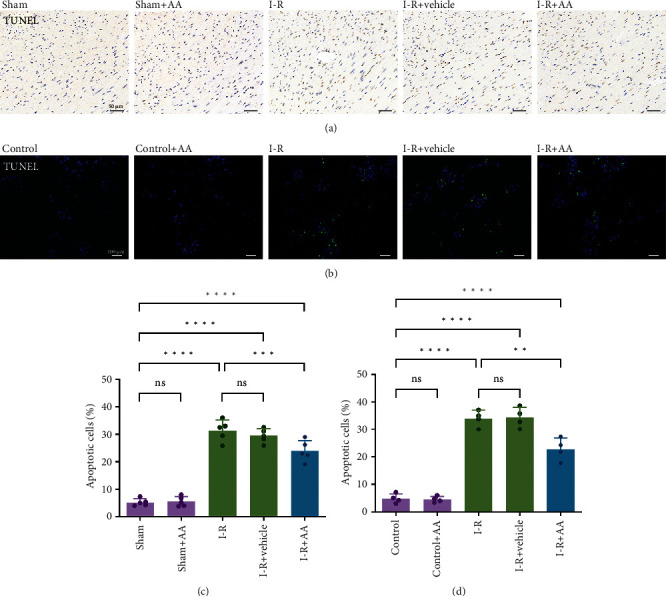
Effects of AA on cell apoptosis. (a) TUNEL-positive cells (deep brown) in left ventricle sections were observed under a microscope (scale bars, 50 *μ*m). (b) TUNEL-positive cells (green fluorescence) in cells were observed under a fluorescence microscope (scale bars, 100 *μ*m). (c) Statistical analysis of apoptosis rate in tissues. (d) Statistical analysis of apoptosis rate in cells. Data represent the mean ± SD (^ns^*P* > 0.05, ^∗∗^*P* < 0.01,  ^∗∗∗^*P* < 0.001, and^∗∗∗∗^*P* < 0.0001). TUNEL: TdT-mediated dUTP nick-end labelling.

Excess oxidative stress and mitochondrial dysfunction will lead to irreversible cell apoptosis and subsequent organ dysfunction [29]. I-R injury has been proven to cause excess ROS production, mitochondrial dysfunction, and uncontrollable apoptosis [30]. Following the AA intervention, the number of TUNEL-positive cells was significantly reduced in vivo (Figures 5(a) and 5(c)) and in vitro (Figures 5(b) and 5(d)), indicating that MIRI-induced apoptosis was alleviated (Figures 5(c) and 5(d)).

**Figure 6:**
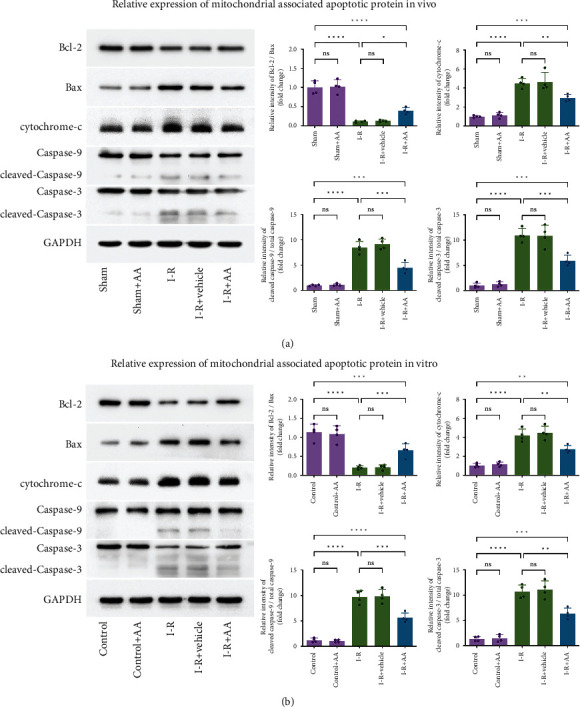
Effects of AA on expression of mitochondrial-associated apoptotic protein. (a) Relative expression of mitochondrial-associated apoptotic protein in vivo. (b) Relative expression of mitochondrial-associated apoptotic protein in vitro. Data represent the mean ± SD (^ns^*P* > 0.05, ^∗^*P* < 0.05,  ^∗∗^*P* < 0.01,  ^∗∗∗^*P* < 0.001, and^∗∗∗∗^*P* < 0.0001).

Apoptosis is strictly regulated in an orderly manner by various signalling pathways [31]. The mitochondria-dependent apoptosis pathway is the main signal transduction pathway involved in the occurrence of apoptosis during I-R injury [29]. Located upstream of mitochondria, Bcl-2 and Bax in the Bcl-2 family are the most important proteins known to regulate apoptosis through the release of cyt-c [32]. Cyt-c is a vital component of the mitochondrial electron transport chain [33]. Its release from mitochondria is the key step in apoptosis, and the activation of caspase cascades depends on the release of cyt-c. According to the analysis of western blot results (Figures 6(a) and 6(b)), the balance of Bcl-2/Bax was disrupted during MIRI, resulting in the substantial accumulation of Bax. Subsequently, the expression level of cyt-c increased, and caspase cascades (caspase-9 and caspase-3) were activated. These results indicate the activation of the mitochondria-dependent apoptosis pathway. We therefore examined the effect of AA on regulating the mitochondria-dependent apoptosis pathway, and the western blot results showed that pretreatment with AA suppressed the expression of mitochondria-dependent apoptosis-related proteins, thereby inhibiting mitochondrial-dependent apoptosis.

### 3.5. p38-MAPK and JNK-MAPK Pathway Engage in the Protective Effects of AA on MIRI-Induced Cell Apoptosis

**Figure 7:**
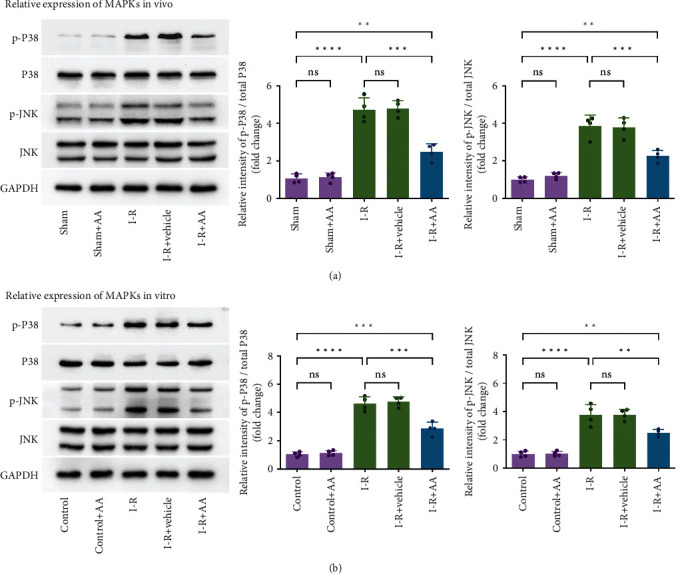
Effects of AA on expression of MAPKs. (a) Relative expression of p38-MAPK and JNK-MAPK in vivo. (b) Relative expression of p38-MAPK and JNK-MAPK in vitro. Data represent the mean ± SD (^ns^*P* > 0.05, ^∗∗^*P* < 0.01,  ^∗∗∗^*P* < 0.001, and^∗∗∗∗^*P* < 0.0001). MAPKs: mitogen-activated protein kinases.

Mitogen-activated protein kinases (MAPKs) are serine/threonine protein kinases. Studies have confirmed that the MAPK signalling pathway plays a vital role in the process of cell biological reactions such as cell proliferation, differentiation, transformation, and apoptosis [34, 35]. p38-MAPK and JNK-MAPK, members of the MAPK family, are extremely sensitive to oxidative stress [36, 37]. Studies have indicated they could affect the mitochondria-dependent apoptosis pathway by acting on Bcl-2 family proteins [38–40]. In the current study, we first examined whether administration of AA affects MIRI-induced activation of the MAPK signalling pathway. As shown in Figures 7(a) and 7(b), consistent with the results of other studies [41, 42], the proteins derived from I-R-treated hearts or NRVMs showed elevated levels of phosphorylated p38-MAPK and JNK-MAPK. In contrast, pretreatment with AA effectively prevented MIRI-induced p38-MAPK and JNK-MAPK phosphorylation.

**Figure 8:**
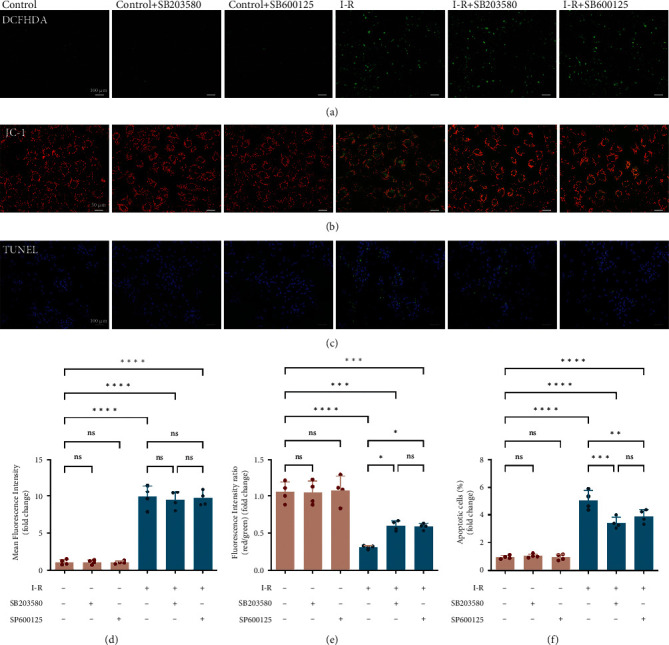
Inhibition of p38-MAPK/JNK-MAPK pathway simulates the protective effects of AA after RIMI stimuli in cardiomyocytes. Cells were preincubated with SB203580 (10 *μ*M) and SP600125 (10 *μ*M) for 1 h before treatment with I-R. (a) Intracellular ROS generation in cells was photographed by DCFH-DA staining (scale bars, 100 *μ*m). (b) MMP in cells was determined by staining with the mitochondrial dye JC-1 (scale bars, 50 *μ*m). (c) TUNEL-positive cells (green fluorescence) in cells were observed under a fluorescence microscope (scale bars, 100 *μ*m). (d) Statistical analysis of ROS generation levels in cells. (e) Statistical analysis of MMP changes in cells. (f) Statistical analysis of apoptosis rate in cells. Data represent the mean ± SD (^ns^*P* > 0.05, ^∗^*P* < 0.05,  ^∗∗^*P* < 0.01,  ^∗∗∗^*P* < 0.001, and^∗∗∗∗^*P* < 0.0001).

Further, to investigate whether the p38-MAPK and JNK-MAPK pathways could be implicated in MIRI induced ROS production, MOMP, and cell apoptosis. SB203580 (p38 inhibitor) and SP600125 (JNK inhibitor) were applied to the NRVMs with the presence or absence of I-R procedure. As shown in Figure 8, pretreatment of cells with SB203580 and SP600125 did not have any effect on ROS production (Figures 8(a) and 8(d)). However, SB203580 and SP600125 effectively reduced the loss of JC-1 red fluorescence (Figures 8(b) and 8(e)) and the rate of apoptosis (Figures 8(c) and 8(f)) induced by I-R, respectively.

Taking all, these data illustrated that the p38-MAPK and JNK-MAPK pathways participate in the cytoprotective effect of AA against MIRI-induced mitochondria-dependent apoptosis.

## 4. Discussion

Based on accumulating evidence, oxidative stress is related to MIRI [11]. The production of a large amount of ROS during MIRI directly or indirectly affects cardiac function, causes cardiomyocyte dysfunction, and promotes cell damage [28, 43]. Mitochondria are the main endogenous source and target of ROS [11]. As the heart is a high energy-consuming organ [44], cardiomyocytes are rich in mitochondria, and thus, cardiomyocytes are very sensitive to oxidative stress signals [45]. Under physiological conditions, a critical balance exists between ROS production and the endogenous antioxidant system [1]. Pathological conditions such as MIRI shift the balance, which is conducive to excess ROS production. This change leads to damage to mitochondrial membrane lipids and activation of the mitochondria-dependent apoptosis pathway to induce cell apoptosis [1, 11, 46].

The main process of the mitochondria-dependent apoptosis pathway is MOMP, which is the result of impaired integrity of the mitochondrial outer membrane (OMM) [47]. MOMP is generally considered an irreversible key step [25]. On the one hand, the occurrence of MOMP causes the release of apoptosis-related proteins into the cytoplasm, which destroys the proton concentration gradient on both sides of the mitochondrial membrane and leads to a decrease in the MMP [48]. The loss of the MMP indicates the opening of the mitochondrial permeability transition pore (MPTP), a landmark event in the early stage of mitochondria-dependent apoptosis [49]. Once the MMP is depleted without intervention, the cell will enter the irreversible stage of apoptosis [9]. On the other hand, cyt-c released during MOMP activates the caspase cascade reaction. This process occurs within a few minutes and rapidly leads to cell apoptosis [50].

The proapoptotic protein Bax and antiapoptotic protein Bcl-2, which belong to the Bcl-2 family of proteins, are considered important regulators of the mitochondria-dependent apoptosis pathway and participate in the initiation of MOMP [51]. The expression of Bax and Bcl-2 in the body is strictly regulated and maintained in a dynamic balance [52]. Bcl-2 is located in membrane systems such as the OMM, endoplasmic reticulum membrane, and outer nuclear membrane, while Bax mostly exists in the cytoplasm in the form of inactive monomers, and a small part of Bax travels between the mitochondria and the cytoplasm. Edlich et al. [53] have shown that in healthy cells, Bax that has been translocated to mitochondria is continuously relocated to the cytoplasm by antiapoptotic proteins such as Bcl-2 on the mitochondria to prevent the occurrence of MOMP. Thus, changes in the Bcl-2/Bax ratio will affect mitochondrial permeability and cell fate [25, 52]. Under stress conditions such as I-R injury, the dynamic balance of Bax between the cytoplasm and mitochondria is broken, Bax accumulates in the OMM and undergoes conformational changes induced by BH3-only proteins [53, 54], destroys the integrity of the OMM, and induces MOMP. The occurrence of MOMP inevitably leads to the loss of the MMP and destroys mitochondrial function, resulting in disrupted ATP synthesis [55, 56]. The lack of ATP synthesis leads to matrix swelling, depolarization of the mitochondria, and finally mitochondrial fragmentation (i.e., mitochondrial division) [27, 46, 57].

The primary members of MAPK signalling pathways have been implicated as proximal effectors of mitochondria-dependent apoptotic mechanisms. In particular, p38-MAPK and JNK-MAPK play pivotal roles in the transmission of apoptotic signals [35]. In cells under oxidative stress, the p38-MAPK/JNK-MAPK pathways are activated and cause upregulation of BH3-only proteins such as Bim and Bid [38–40], which function to promote the conformational change and translocation of Bax to the mitochondria, triggering the mitochondria-dependent apoptosis pathway.

**Figure 9:**
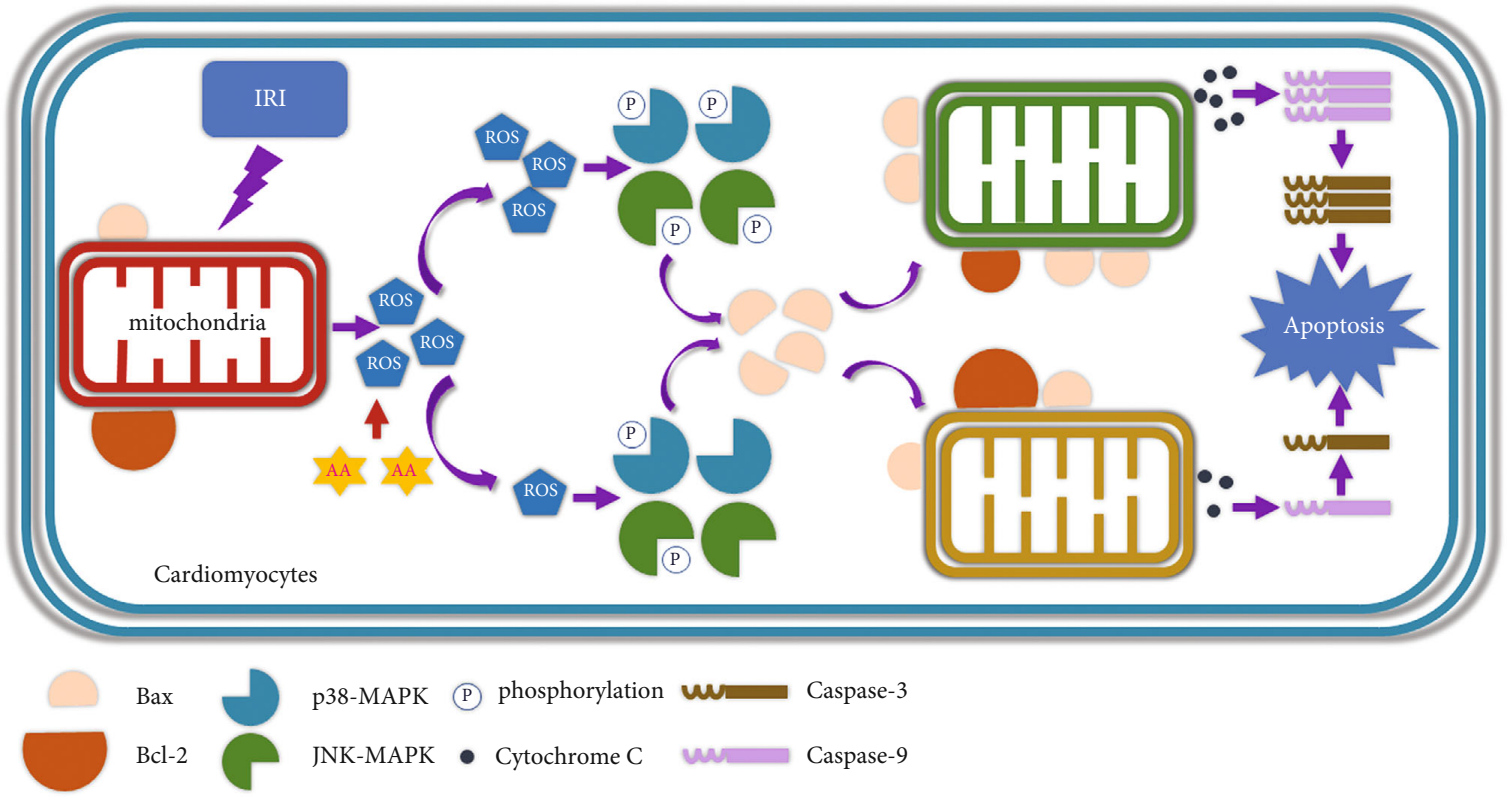
Schematic illustration of the current study. By targeting mitochondrial ROS generation, AA reduces ROS-mediated MAPK phosphorylation, balances the proportion of Bcl-2 and Bax, maintains MMP, and inhibits mitochondrial MOMP. Thus, the release of cyt-c and the activation of caspase cascades were blocked, eventually reducing cell apoptosis caused by I-R. MOMP: mitochondrial outer membrane permeabilization; cyt-c: cytochrome c; I-R: ischemia/reperfusion.

As a traditional Chinese medicine, AA exerts beneficial effects on oxidative stress in many disease models. In our previous study, we found that AA reduced ROS generation during MIRI and inhibited excess autophagy [18]. The results of the current study suggest that oxidative stress-induced injury plays a significant role in MIRI. Mitochondrial dysfunction is the central cause of injury [58]. Copious amounts of accumulated ROS activate the p38-MAPK/JNK-MAPK signalling pathway and alter Bcl-2 family proteins through direct or indirect effects. These changes lead to a serious imbalance between proapoptotic proteins and inhibitory proteins on the mitochondrial membrane, causing mitochondrial depolarization and MOMP and eventually activating the mitochondria-dependent apoptosis pathway. AA exerts a protective effect on MIRI by improving the cardiac function and reducing cardiomyocyte damage. Due to its antioxidant properties, AA alleviates mitochondrial oxidative stress, as evidenced by the stable mitochondrial structure, maintained MMP, and reduced ROS generation. Otherwise, due to its antiapoptotic properties, AA inhibits the MAPK/mitochondria-dependent apoptosis pathway, as evidenced by the limited phosphorylation of p38-MAPK and JNK-MAPK, balanced ratio of Bcl-2/Bax, reduced cyt-c release, inhibition of caspase cascade, and reduced apoptosis (Figure 9).

In recent decades, considerable progress has been achieved in treatments designed to preserve the function of mitochondria and reduce oxidative stress during MIRI in vitro and in vivo. However, one of the greatest challenges is to transform the efficacy of preclinical animal research into clinical practice [1]. Since AA has been used in clinic [59, 60], AA has potential clinical application value in the treatment of CHD.

## 5. Conclusions

Our findings demonstrated that AA effectively protects cardiomyocytes against MIRI by inhibiting the ROS-mediated MAPK and mitochondria-dependent apoptosis pathway (Figure 9). The results suggest that AA is a promising therapeutic drug for CHD patients, especially in the situation of AA which has been applied clinically.

## Data Availability

The datasets used and/or analyzed during the current study are available from the corresponding author on reasonable request.

## Supplementary Materials

Supplementary Materials Supplementary Figure: (A) chemical structure of AA. (B) The cytotoxicity of AA on NRVMs was assessed via the CCK-8 assay. 20 *μ*M was chosen as the dose of AA for the following study. Data represent the mean ± SD (*n* = 3) (^∗^*P* < 0.05,  ^∗∗^*P* < 0.01, and^∗∗∗^*P* < 0.01).
