# Supplementary figures and images for: Asiatic Acid Alleviates Myocardial Ischemia-Reperfusion Injury by Inhibiting the ROS-Mediated Mitochondria-Dependent Apoptosis Pathway
Source: Oxid Med Cell Longev. 2022 Feb 14;2022:3267450. doi: 10.1155/2022/3267450 (PMC8860531; doi:10.1155/2022/3267450)

**A**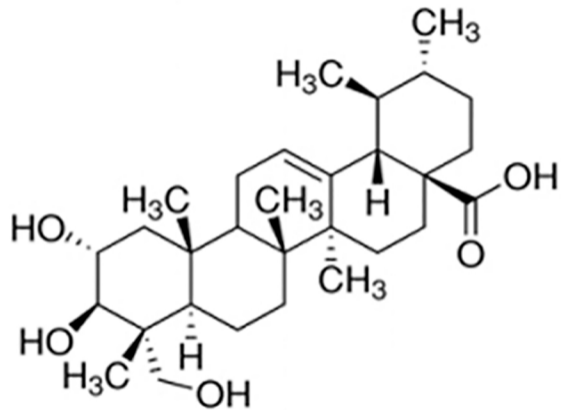**B**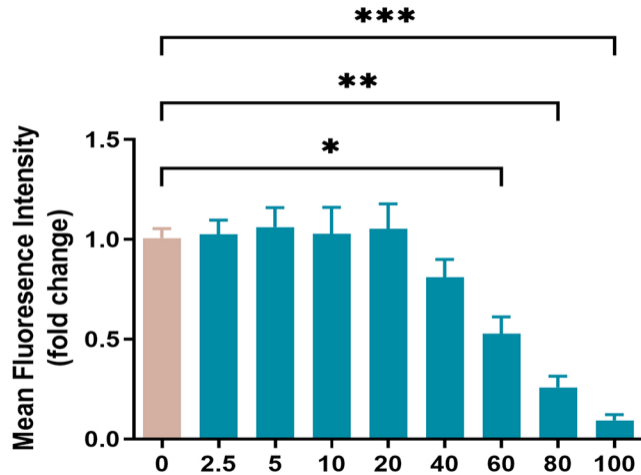

Supplement: Supplementary Materials — Supplementary Figure: (A) chemical structure of AA. (B) The cytotoxicity of AA on NRVMs was assessed via the CCK-8 assay. 20 μM was chosen as the dose of AA for the following study. Data represent the mean ± SD (n = 3) (∗P < 0.05, ∗∗P < 0.01, and∗∗∗P < 0.01). [file 3267450.f1.pdf]
